# Metabolome and Transcriptome Sequencing Analysis Reveals Anthocyanin Metabolism in Pink Flowers of Anthocyanin-Rich Tea (*Camellia sinensis*)

**DOI:** 10.3390/molecules24061064

**Published:** 2019-03-18

**Authors:** Dylan O’Neill Rothenberg, Haijun Yang, Meiban Chen, Wenting Zhang, Lingyun Zhang

**Affiliations:** 1 College of Horticulture Science, South China Agricultural University, Guangzhou 510640, China; Dylan.Rothenberg@colorado.edu (D.O.R.); meibanchen@163.com (M.C.); wendyzhang998@163.com (W.Z.); 2 Center of Experimental Teaching for Common Basic Courses, South China Agricultural University, Guangzhou 510640, China; hjyang@scau.edu.cn

**Keywords:** *Camellia sinensis* (L.), metabolome, transcriptome, flower development, anthocyanin-rich cultivar, anthocyanin biosynthesis, WGCNA

## Abstract

Almost all flowers of the tea plant (*Camellia sinensis*) are white, which has caused few researchers to pay attention to anthocyanin accumulation and color changing in tea flowers. A new purple-leaf cultivar, Baitang purple tea (BTP) was discovered in the Baitang Mountains of Guangdong, whose flowers are naturally pink, and can provide an opportunity to understand anthocyanin metabolic networks and flower color development in tea flowers. In the present study, twelve anthocyanin components were identified in the pink tea flowers, namely cyanidin *O*-syringic acid, petunidin 3-*O*-glucoside, pelargonidin 3-*O*-beta-d-glucoside, which marks the first time these compounds have been found in the tea flowers. The presence of these anthocyanins seem most likely to be the reason for the pink coloration of the flowers. Twenty-one differentially expressed genes (DEGs) involved in anthocyanin pathway were identified using KEGG pathway functional enrichment, and ten of these DEG’s screened using venn and KEGG functional enrichment analysis during five subsequent stages of flower development. By comparing DEGs and their expression levels across multiple flower development stages, we found that anthocyanin biosynthesis and accumulation in BTP flowers mainly occurred between the third and fourth stages (BTP3 to BTP4). Particularly, during the period of peak anthocyanin synthesis 17 structural genes were upregulated, and four structural genes were downregulated only. Ultimately, eight critical genes were identified using weighted gene co-expression network analysis (WGCNA), which were found to have direct impact on biosynthesis and accumulation of three flavonoid compounds, namely cyanidin 3-*O*-glucoside, petunidin 3-*O*-glucoside and epicatechin gallate. These results provide useful information about the molecular mechanisms of coloration in rare pink tea flower of anthocyanin-rich tea, enriching the gene resource and guiding further research on anthocyanin accumulation in purple tea.

## 1. Introduction

Tea (*Camellia sinensis* (L.)) is an important economic plant in south China and Southeast Asia. Tea contains bioactive flavonoids, and therefore has received much attention due to it’s beneficial health attributes. Tea has been found to have beneficial effects on both physical health [1,2,3] and cognition [4,5]. Flavonoids, including anthocyanidins, flavan-3-ols, flavonols, flavones, flavanones and isoflavones, are important water-soluble pigments, which are plant secondary metabolites [6]. These pigments help to form the bitterness and astringency of tea flavor, and due to their antioxidant activities, may be used to prevent chronic diseases including cardiovascular [7] and inflammatory diseases [8]. In recent years, many researchers have been attracted to plant anthocyanins due to their notable antioxidant capacity [9].

Anthocyanin-rich tea leaves enhance antioxidant properties of the final tea product, however studies in the past have shown that green tea produced using anthocyanin-rich purple tea leaves is of inferior taste quality, due to evident bitterness and astringency [10]. In recent years, researchers have studied anthocyanin-rich tea cultivars in order to better understand anthocyanin accumulation in tissues, and molecular biological mechanisms behind color formation. Anthocyanin composition and content in anthocyanin-rich purple tea cultivars have been determined by numerous researches [11,12,13]. Composition and ratio of anthocyanins are responsible for differences in leaf color, and physiological and biochemical processes [14].

Recently, several particularly anthocyanin-rich purple-leaf tea varieties have been developed in different countries [15,16,17]. There have been eight anthocyanins isolated and identified from purple tea, and their health functions have been preliminarily studied [18,19]. Important molecular mechanisms responsible for differentiating purple-leaf tea cultivars have been briefly elaborated on. Studies have used various biotechnologies to elucidate structural genes and regulating factors involved in anthocyanin accumulation in these tea plants [11,12,13,14,20,21,22,23].

In the flavonoid biosynthesis pathway, cinnamoyl-(C_6_-C_3_) is combined with three malonyl-CoA units to yield the backbone. Studies have used model species to confirm functional and regulatory genes involved in the flavonoid biosynthesis pathway. Two main classes of functional enzymes exist in this pathway; “early biosynthetic step” enzymes, which catalyze all flavonoid synthesis, and “later biosynthetic step” enzymes, which are required for synthesis of flavonols and anthocyanins [24,25]. However, the relationships among these plant flavonoids still remain unclear. This is due to competition for branches and substrate within the flavonoid biosynthetic pathway. For example, a similar biosynthetic pathway is often used to produce specialized metabolites in reproductive and trophic tissues of plant, however, to date, flavonoid biosynthesis in vegetative tissue and that in reproductive tissue have not been shown to correlate strongly with one another. For example, regardless of flower color, most species synthesize only common flavonols, (i.e., quercetin and kaempferol) in leaves, and rarely are anthocyanins found. This seem to indicate that changes in flower color may occur independently of, or without significantly relation to, the accumulation of flavonoids in vegetative tissue [26].

In the case of tea (*Camellia sinensis L*.), the flowers have always been observed to be white in color, including those of purple-leaf cultivars, such as Zijuan and Zixin. However, our researchers recently found a natural mutant variety of purple-leaf tea, which also has pink flowers, and a higher anthocyanin content compared to common cultivars. This novel cultivar, which we are calling Baitang purple tea (BTP), offers us a new perspective on the metabolomics behind anthocyanin accumulation in plant tissue. The production of anthocyanins in both the vegetative and reproductive tissue of BTP may provide rare evidence that flavonoid biosynthesis within these two tissues can be correlated, and perhaps regulated by the same key genes.

In most anthocyanin-rich tea leaves, these metabolites function to protect against strong sun exposure in summer months. However, flowers of tea (*Camellia sinensis* L.) bloom in winter, meaning that anthocyanin synthesis in BTP flowers must be serving a different function than in vegetative tissue. As leaves of BTP lose anthocyanins with colder weather, becoming less purple, the budding flowers synthesize anthocyanin and gain purple coloration. We hypothesize this is an adaptation to mitigate against cold-stress damage inflicted upon reproductive tissue during winter months. However, we remain uncertain of the relationship between anthocyanin synthesis in vegetative tissue and that in reproductive tissue, because anthocyanin biosynthesis in tea flowers has never been researched prior to this study. Anthocyanin synthesis in the flowers of other *Camellia* plants, such as *Camellia nitidissma* and *Camellia chekiangoleosa* have been studied in the past, however a lack of genomic information and developed genomic tools has left key links in the metabolic pathway unclear.

Understanding key genes involved in activating floral anthocyanin synthesis could be valuable for several reasons. First, if our cold-stress hypothesis is correct, there might be value in transferring these genes into tea plants of growers at higher latitudes who have experienced high incidents of plant damage or death in recent years due to extreme frost conditions resulting from climate change. Stronger natural plant defenses for winter conditions could serve to mitigate damages at low economic and environmental cost. On the consumer side, this study has revealed three previously unknown anthocyanins (cyanidin *O*-syringic acid, petunidin 3-*O*-glucoside, and pelargonidin 3-*O*-beta-d-glucoside), which might offer added taste benefits due to their attached glycosides, in addition to the sizeable number of health benefits already attributed to anthocyanin compounds. Furthermore, other *Camellia* plants with high ornamental value, such as *Camellia nitidissma*, *Camellia chekiangoleosa,* and *Camellia japonica*, all highly selected for in landscaping due to the rarity of winter-blooming ornamental flowers, may benefit from a greater understanding of the key genes involved in metabolic processes behind their color formation. This is not to mention that rare purple tea flowers themselves may serve great ornamental value in their own right. Thus, the current study aims to use biological networks tools to analyze the genomic data sets of BTP flowers at various stages of development in order to understand the key genes and metabolic activity driving BTP’s unique phenotypic traits.

The primary biological networks analysis tool used in this research was weighted gene co-expression network analysis (WGCNA). This advanced genomic network analysis tool uses a framework and network language that surpasses standard analysis techniques. WGCNA assesses high-dimensional data sets in order to find clusters of interconnected data that reflect network interrelation and connectivity. The program identifies clusters of interrelated genes (which it calls “modules”), thus facilitating the identification of co-expression of modules with correlating phenotypes. In this study, WGCNA identified genes with significant co-expression with the biosynthesis of metabolites in the anthocyanin pathway we believe to be responsible for the unique phenotype observed in BTP flowers.

In the current study, our aim was to identify significantly differentially expressed genes involved in anthocyanin biosynthesis during tea flower development. To do this, we first conducted metabolic profiling and transcriptome analysis at five progressive stages of tea flower development in order to focus on anthocyanin biosynthetic pathways. Pink flowers of BTP (*Camellia sinensis* L.) were monitored for candidate genes, particularly focusing on structural genes involved in anthocyanin biosynthesis and the identification of differentially expressed genes (DEGs). Critical structural genes involved in anthocyanin biosynthesis were screened using functional enrichment. Additionally, co-expressed genes relating to unique components of anthocyanin synthesis were identified using weighted gene co-expression network analysis (WGCNA) during the pink tea flower development.

## 2. Results

### 2.1. Metabolomic Differences among Tea Flower Development Stages

**Figure 1:**
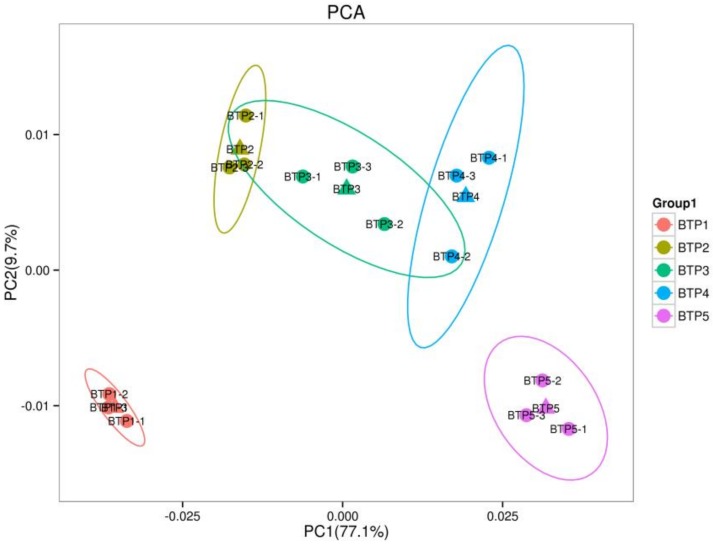
PCA scores plot of different floral development stages by UPLC-ESI-MS/MS metabolite analysis. PC1, principal component 1; PC2, principal component 2. Explained variants PC1: 77.1%, PC2: 9.7%.

In this study, metabolic profiling of the pink tea flowers using an UPLC-ESI-MS/MS system identified 638 metabolites. Metabolite profiles of pink tea flowers were then subjected to principal component analysis (PCA). The score plots of PCA (Figure 1) exhibited an obvious separation between different flower development stages. The first two principal components (PC1 and PC2) for the flowers were 77.1% and 9.7%, respectively. The large PC1 value indicates a large degree of genetic variance based on development stage. However, two intersectional points found between BTP2/BTP3 and BTP3/BTP4 indicated that the metabolic profile was more similar among stages 2, 3, and 4, than between the first and fifth stages, whose PCA score clusters were clearly differentiated with no points of overlap. Altogether, the PCA clusters indicate that the first stage, the fifth stage and the overlapping second, third, and fourth stages define three subgroups—additionally (and surprisingly) that the first and the fifth stages are most distinct.

**Figure 2:**
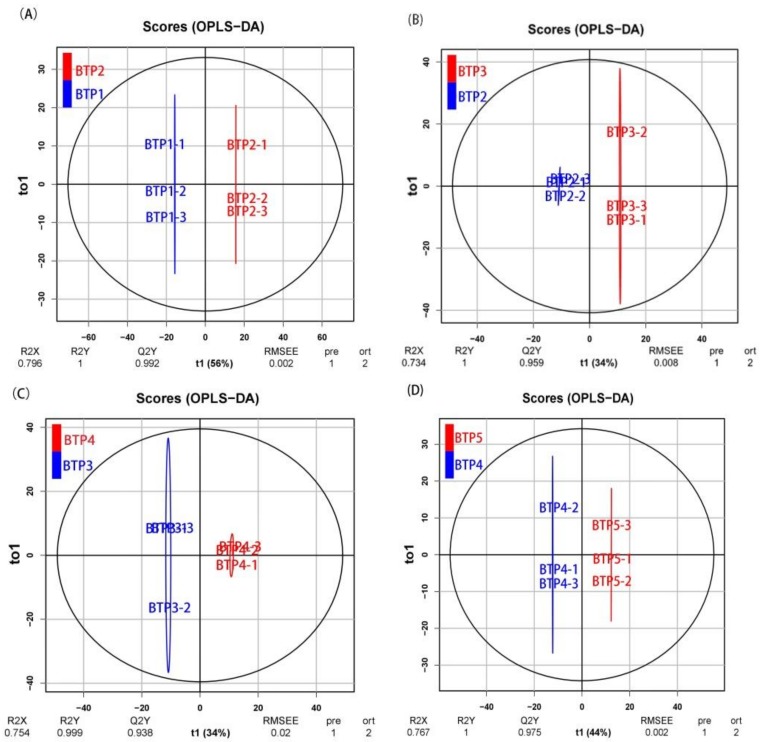
OPLS-DA score plots demonstrating the clustering pattern of metabolites in subsequent development stages of tea flowers. X-axes represent PC1, Y-axes represent PC2; RMSEE represent Root Mean Square Error of Estimation. (**A**) BTP1 vs BPT2 group (R2Y  =  1, Q2Y  =  0.992, RMSEE  =  0.002 ); (**B**) BTP2 vs BPT3 group (R2Y  =  1, Q2Y = 0.959, RMSEE  =  0.008); (**C**) BTP3 vs BPT4 group (R2Y  =  0.999, Q2Y  =  0.938,RMSEE = 0.02); (**D**) BTP4 vs BPT5 group (R2Y  =  1, Q2Y  =  0.975,RMSEE  =  0.002).

The OPLS-DA results indicated that the main biological components were significantly changed along with changing stages of development (Figure 2). Figure 2 is an OPLS-DA plot showing the significance of change in metabolic profile moving from one development stage to the next. Figure 2 shows the composition between the first stage and second stage (BTP1 vs. BTP2), second stage and third stage (BTP2 vs. BTP3), third stage and fourth stage (BTP3 vs. BTP4) and fourth stage and fifth stage (BTP4 vs. BTP5) respectively clustered together in the OPLS score plots. The R2Y of this OPLS-DA model was 1.0, 1.0, 0.999 and 1.0 in metabolomic differences of different floral development, respectively. While, the Q2Y of the model was 0.992, 0.959, 0.938 and 0.975 (from BTP1 to BTP5), respectively. These data demonstrate highly significant differences in metabolite profiles based on development stage.

Altogether, twelve anthocyanins were identified from BTP flowers, including procyanidin B2, procyanidin B3, pelargonidin 3-*O*-beta-d-glucoside, pelargonin, cyanidin, cyanidin 3,5-*O*- diglucoside, cyanidin *O*-syringic acid, luteolin *O*-hexosyl-*O*-hexosyl-*O*-hexoside, cyanidin 3-*O*-rutinoside, peonidin, petunidin 3-*O*-glucoside, and malvidin 3,5-diglucoside (Supplementary Table 1). Excluding procyanidins, the predominant anthocyanins were found to be cyanidin and its glycoside-derived compounds, namely cyanidin 3,5-*O*-diglucoside, cyanidin 3-*O*-glucoside, and cyanidin *O*-syringic acid. Among the anthocyanin compounds discovered in BTP flowers, 3 had never before been discovered in *Camellia sinensis L.* These novel anthocyanin compounds include pelargonidin 3-*O*-beta-d-glucoside, petunidin 3-*O*-glucoside, and cyanidin *O*-syringic acid. Two of the three novel anthocyanin compounds were also among the compounds found in particularly high concentration in BTP flowers, suggesting that these two compounds (cyanidin *O*-syringic acid and petunidin 3-*O*-glucoside) may be instrumental in explaining the unique phenotype of BTP flowers.

### 2.2. Overview of Transcriptome Sequencing

RNA from fifteen tea flower samples was used for RNA-seq with three replicates per flower development stage. Before cDNA libraries were subjected to pair-end reading with the Illumina Hiseq 2500 system, the low quality reads and adapter sequences were removed. Thus, between 20, 915, 131 and 28,163,774 clean reads remained (Supplementary Table S3). All clean reads were subsequently matched to the reference *Camellia sinensis var. Assamica* genome databases. Ultimately, most sequences from BTP1 (75.81–76.42%), BTP2 (75.52–76.29%), BTP3 (76.27–77.22%), BTP4 (75.37–77.81%) and BTP5 (75.90–76.68%) were matched to the reference genome. The numbers of reads mapped to ‘+’ and ‘−‘ strands were nearly equal in different flower development stages (Supplementary Table S4).

### 2.3. Annotation and Identification of Unigenes

**Figure 3:**
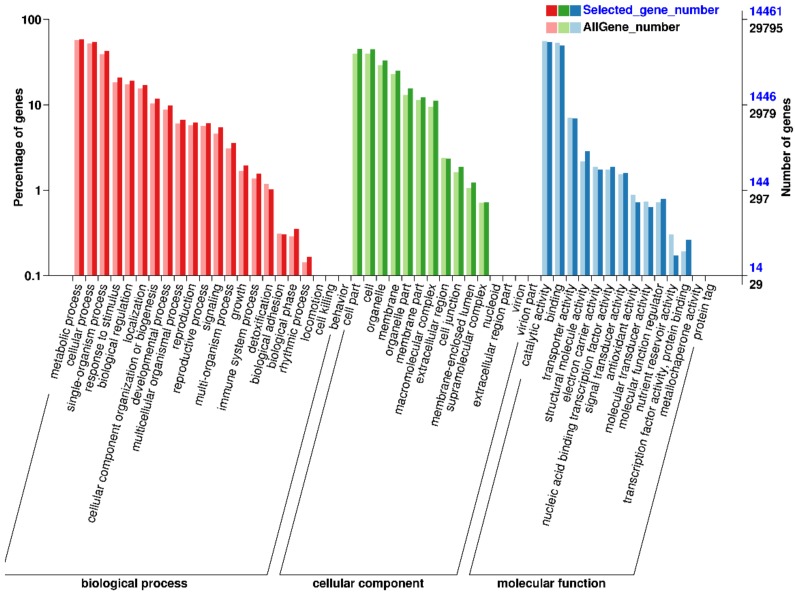
GO classification of unigenes from pink *Camellia sinensis* flower.

All the assembled unigenes were annotated using BLASTx against the public databases: GO, Pfam, Swiss-Prot, Nr, Nt, KO, KOG/COG and KEGG. Assembled unigenes were subjected to GO analysis using Blast-GO (functional categories as shown Figure 3). A total of 29,795 unigenes were categorized into 51 functional groups according to the GO database. The three main three functional categories were biological process, cellular component and molecular function (Figure 3). Among the functional groups, “metabolic process” (17,200 unigenes, GO:0008152, 57.73%) was the most abundant group (Figure 3, Supplementary Table S1).

**Figure 4:**
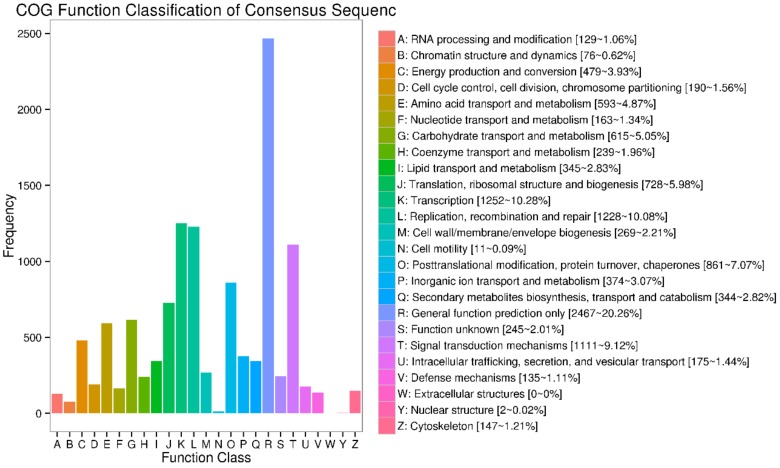
Classification of unigenes from pink *Camellia sinensis* flower.

The assembled unigenes were searched against the COG protein database to classify and estimate probable functions. In all, 13,740 unigenes were clustered into twenty-five functional categories (Figure 4). Among these categories, the “general function prediction only” (R, 2467 unigenes) accounted for 20.26%, was the largest amount. “General functional prediction only” is a category for which only a general prediction regarding the function of that protein is feasible (e.g., that of biochemical activity). A significant majority of the COG’s could be assigned to a well-defined functional category, however the fact that the largest category remains unspecific in nature indicates a degree of ignorance within the COG database. The next largest category was transcription (K, 11,252 unigenes, 10.28%), replication, recombination and repair (L, 1228 unigenes, 10.08%), signal transduction mechanisms (T, 1111 unigenes, 9.12%). The smallest group was nuclear structure (Y, 2 unigenes, 0.02%). The COG and GO annotations showed that unigenes expressed in tea flowers encode various proteins related to metabolism.

### 2.4. KEGG Pathway Analysis

In order to further identify the metabolic pathways involved in the flavonoids’ formation process, all unigenes were searched against the KEGG database. In total, 6041 unigenes were identified and annotated to 127 KEGG pathways (Supplementary Figure S1, Supplementary Table S2). Results showed that most enriched was the metabolic pathway, followed by genetic information processing. Less enriched than these two pathways were organismal systems, environmental information processing, and cellular processes. When we focused on anthocyanin accumulation pathway in tea flower development, 118, 55 and 8 unigenes were annotated according to KEGG pathway enrichment analysis, which involved the phenylpropanoid, flavonoid and anthocyanin metabolism pathways respectively. Phenylpropanoids is an important group of phenyl-alanine-derived physiologically active secondary metabolites in plant. From *p*-coumaroyl-CoA, anthocyanins, flavonols, isoflaconoids and flavonoids share the same phenylpropanoid metabolism pathway for their synthesis. The three pathways mentioned above have a direct bearing on the coloration of tea flower, and offered useful information for identifying DEGs, which encoded proteins that ultimately determined flower color via biosynthesis of anthocyanin.

### 2.5. Screening of Differentially Expressed Genes during Floral Development

**Figure 5:**
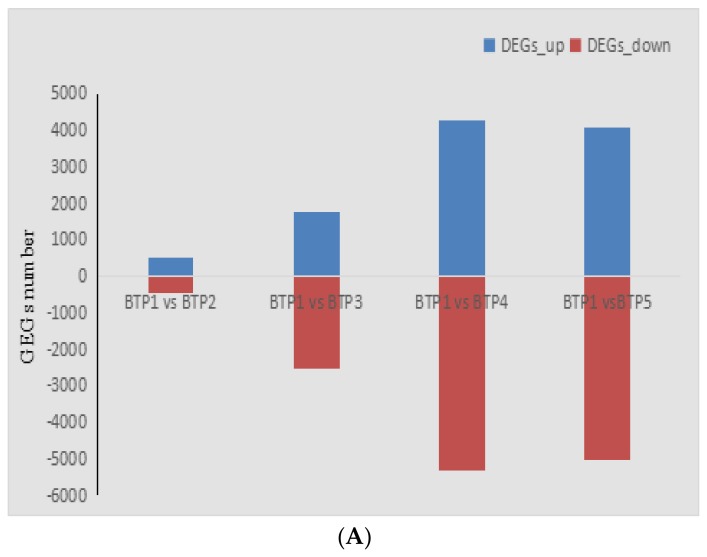

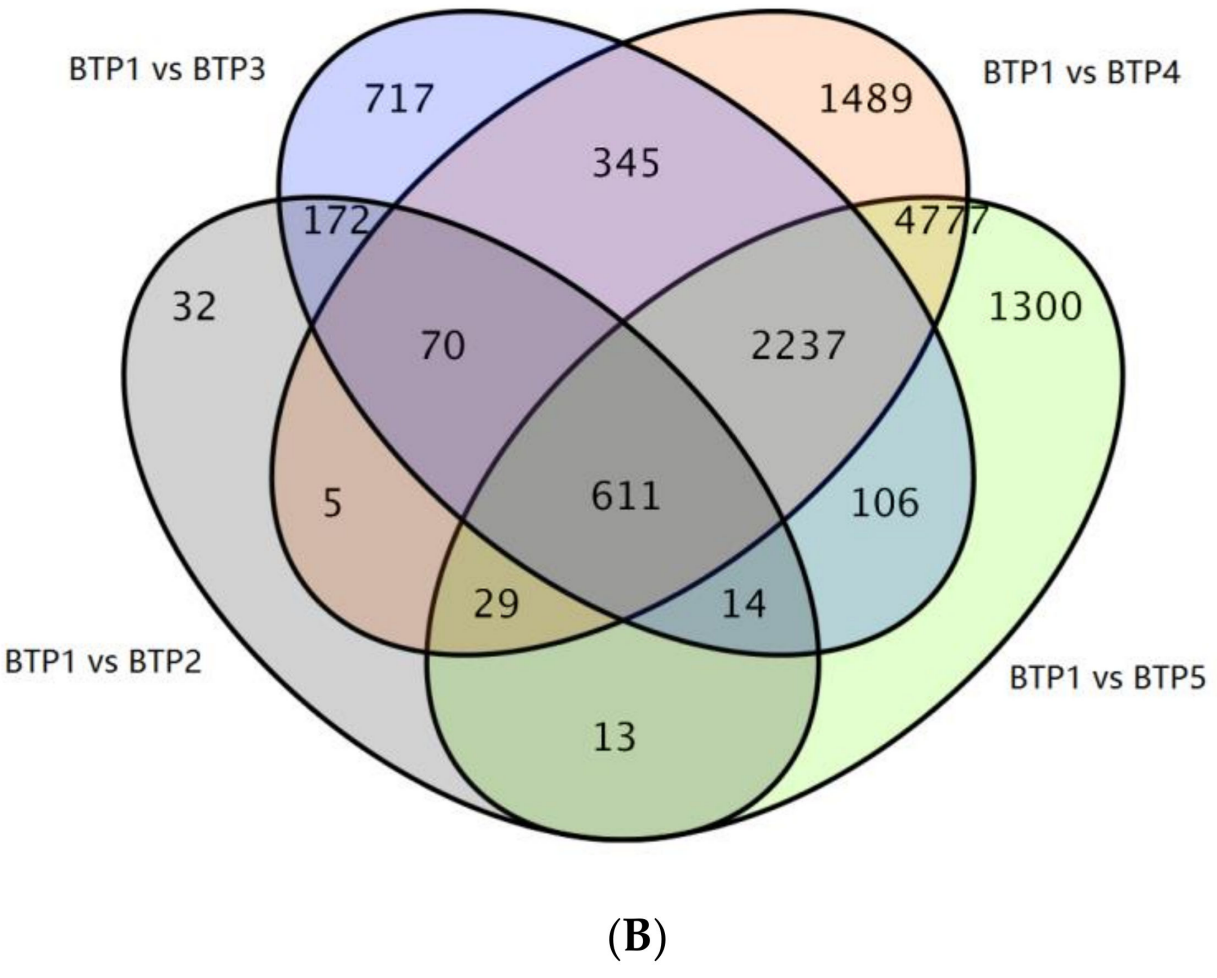
Differentially expressed genes at various stages of BTP flower development (**A**) DEGs among BTP1 vs. BTP2, BTP1 vs. BTP3, BTP1 vs. BTP4, and BTP1 vs. BTP5. Blue: up–regulated expression of DEGs, Red: down–regulated. (**B**) Venn Diagram of DEGs between 4 groups.

We compared the differentially expressed genes (DEGs) in pink tea petals among different floral development stages. In total, 23,868 DEGs were identified, which were differentially expressed at different tea flower development stages. The number of differentially expressed genes had very high variance among different development stages. The largest number of DEGs was found between BTP1 and BTP4, where 9563 DEGs were identified. The libraries of BTP1 vs. BTP2, BTP1 vs. BTP3, BTP1 vs. BTP4, and BTP1 vs BTP5 respectively had 946, 4272, 9563 and 9087 DEGs. Among those DEGs, the four libraries had 611 common DEGs. There were 513 upregulated DEGs in BTP1 vs. BTP2, 1763 upregulated DEGs in BTP1 vs. BTP3, 4257 upregulated DEGs in BTP1vs. BTP4, and 4062 upregulated DEGs in BTP1vs. BTP5 (Figure 5A,B, Supplementary Table S5). These results indicated that the fourth developmental stage of tea flower was the most active in up-regulating the anthocyanin biosynthetic pathway, and perhaps most responsible for anthocyanin biosynthesis compared with other developmental stages. Stage 4 also had the most dramatic change of enzymes and biochemical metabolism across multiple metabolic pathways.

In order to confirm the DEGs involved in metabolism pathways at tea flower development stages, we used tools at the KEGG database to identify DEG-enriched pathways [27]. According to the KEGG enrichment analysis, metabolic activities related to plant development were the most active pathways, such as hormone signal transduction, and starch and carbon metabolism. Notably, genes related to phenylpropanoid biosynthesis were also enriched in all floral development stages. The flavonoid biosynthesis pathway involved in floral petal coloration among four pairwise comparisons included 10, 21, 32, and 31 DEGs in BTP1 vs. BTP2, BTP1 vs. BTP3, BTP1 vs. BTP4, and BTP1 vs. BTP5, respectively (Supplementary Table S3).

### 2.6. Differential Expression Involved in Anthocyanins’ Biosynthetic Genes in Five Floral Development Stages

**Figure 6:**
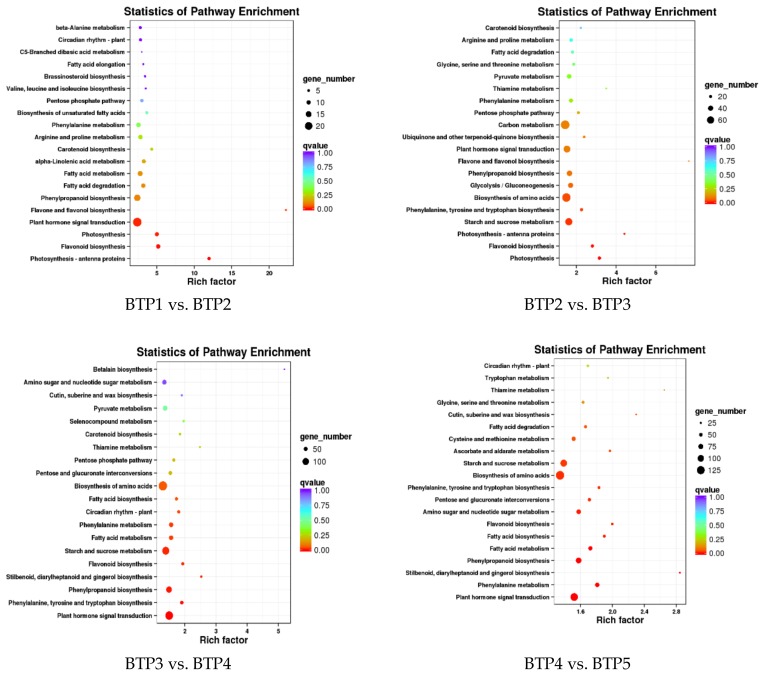
KEGG enrichment analysis of DEGs in different tea development stages.

As an important metabolic branch of the flavonoid pathway, anthocyanin biosynthetic pathway is responsible for the production of anthocyanins in different plant tissues. Paralogue “color” genes and their expression characteristics have been widely researched, including chalcone synthase (CHS), chalcone isomerase (CHI), flavanone-3-hydrozylase, flavonoid 3′-hydroxylase (F3’H) and flavonoid 3′,5′-hydroxlase (F3′5′H). By comparing different developmental stages of BTP flowers using venn analysis and KEGG pathway functional enrichment (Figure 6), we identified these critical pathways and genes relating to anthocyanin biosynthesis. Genes involved in flavonoid and anthocyanin metabolism were screened out using biocloud analysis network platform.

We mentioned that CHS is a pivotal enzyme for the biosynthetic pathway of anthocyanins and flavonoids [28]. In present study, six CHS genes were identified by functional enrichment analysis, and among them, three genes were highly expressed (FPKM values >100), CSA029707, CSA024718, CSA029773. One CHS gene, CSA031662 was almost undetectable from the second stage to the fifth stage of tea floral development, while CSA024718 was most highly expressed across all tea floral development stages (FPKM values > 1000).

Previous research showed chalcone isomerase (CHI) played an important role in formation of the flavanone precursors and floral pigments. By inducing the catalysis of chalcone into naringenin, CHI directly influences the formation of chalcone and anthocyanin [29]. Two CHI genes were identified in BTP flower development, CSA008261 being more highly expressed. Our study annotated two F3H genes, responsible for mediating the conversion of naringenin into dihydrokaempferol.

Of the two F3H genes, CSA004930 was the most abundantly expressed. Two F3′H genes (CSA023049 and Csng5156) were found in our dataset, with CSA023049 expressed relatively highly. The encoded F3′H converts dihydrokaempferol into dihydroquercetin, while F3′5′H converts dihydroquercetin into dihydromyricetin. These compounds are all involved in synthesis of delphinidin-based anthocyanins, which directly relate to flower color formation [30,31]. In our study, two F3′5′H genes were annotated, CSA031792 being the more highly expressed of those in early floral stages.

**Figure 7:**
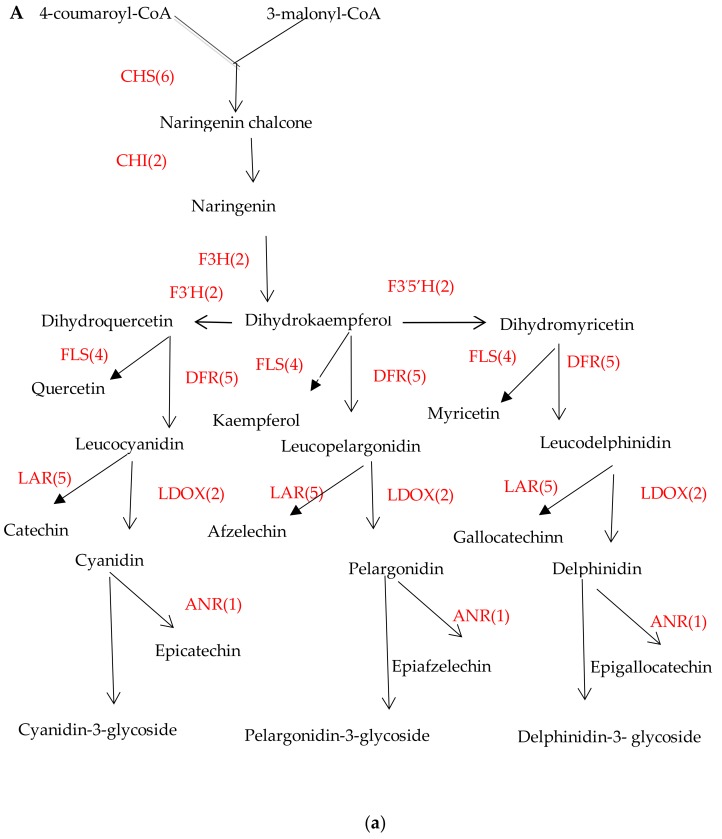

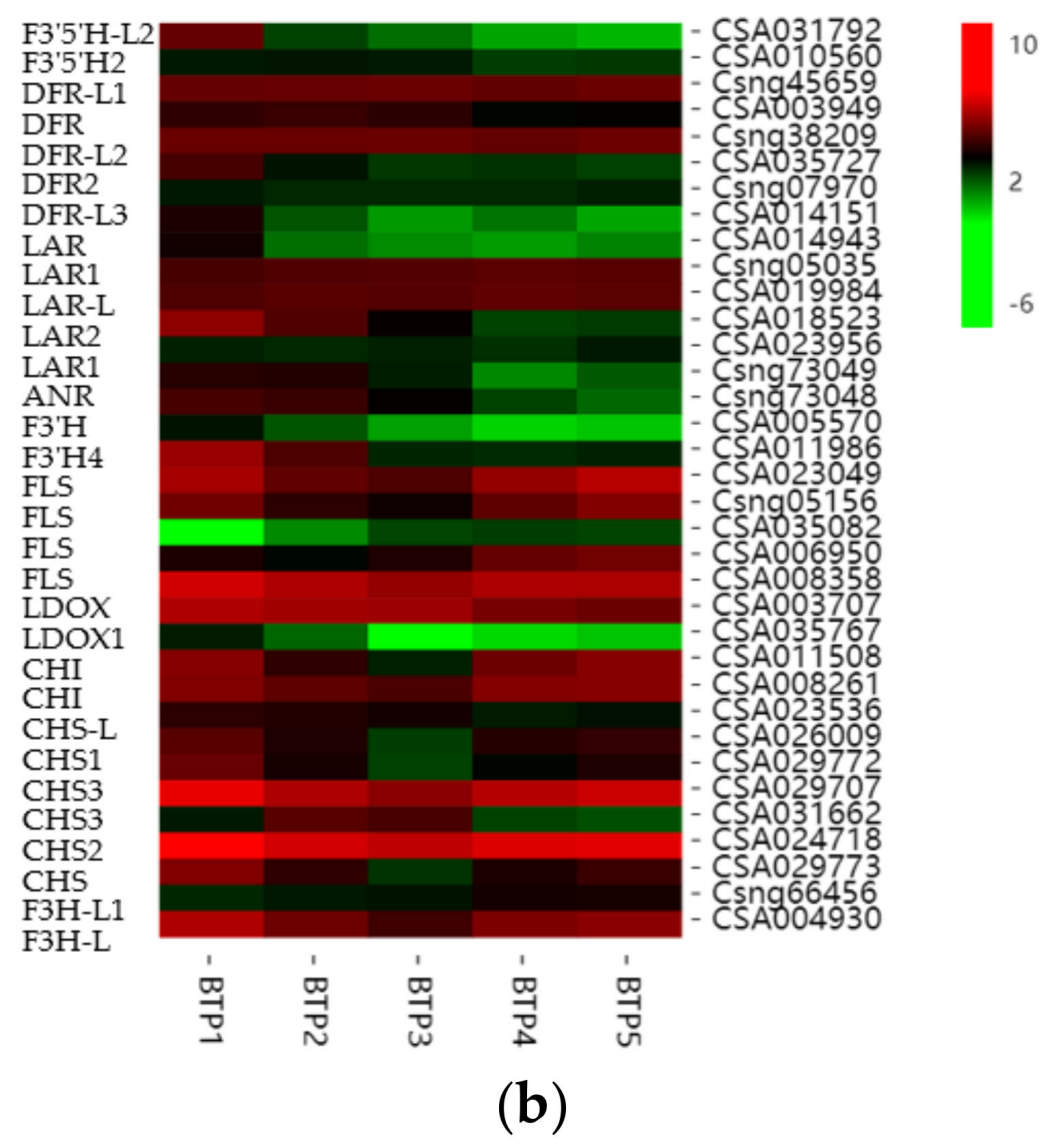
Key structural genes and their expression level of involved in anthocyanin biosynthesis pathway. (**A**) the number of DEGs involved in anthocyanin biosynthetic pathway. (**B**) The heatmap of key structural genes and expression level. The color scale represents the log2 values (FPKM) of the tea flower during the BTP1, BTP2, BTP3, BTP4, and BTP5 stages (from left to right).

Anthocyanins and flavonols are products of the phenylpropanoid metabolic pathway (Figure 7A), in which both compounds are made from dihydroflavonol through two different steps catalyzed by dihydroflavonol 4-reductase (DFR) or flavonol synthase/flavanone 3-hydroxylase(FLS), respectively. DFR involved in anthocyanin biosynthesis catalyzes the conversion of dihydroquercetin into leucoanthocyanins, and functions as a key rate-limiting enzyme [32]. FLS converts dihydroflavonols into flavonols, and plays a competing role with DFR at a crucial branch point in the anthocyanin pathway [32]. Colorless or yellowish flavonols can be formed from dihydroflavonols by FLS, one of the 2-ODD class of enzymes [33]. The endogenous FLS mRNA transcript occurs in early stages of petal development, concurrent with accumulation of flavonols in bud tissue, and terminates at the start of anthocyanin production [34]. In the current study, five DFR genes involved in anthocyanin pathway were identified. Among those five, two were new unigenes annotated as DFR (Csng38209, Csng45659), both of which had relatively highly expression levels. Four FLS genes were identified, with CSA008358 being the most highly expressed (FPKM value > 924), and CSA003707 being relatively highly expressed among all stages (FPKM value from 68 to 330), which likely caused more biosynthesis of flavonols and less biosynthesis of anthocyanin due to competition for substrate.

Competing with FLS and jointly acting with DFR, leucoanthocyanidin dioxygenase (LDOX, or anthocyanidin synthase, ANS) can use dihydroflavonols to synthesize anthocyanidins or proanthocyanidins (PAs) [35]. However, anthocyanins and PAs can be converted into catechins by LAR [36]. In our study, two LDOX genes (CSA035767 and CSA011508) were found in our dataset, with CSA011508 being relatively highly expressed. Five LAR genes (CSA014151, CSA014943, CSA019984, CSA018523 and Csng5035) were found in our dataset, with CSA018523 being relatively highly expressed in initial first stages of floral development (FPKM value > 149).

When we made comparisons among four groups consisting of BTP1 vs. BTP2, BTP2 vs. BTP3, BTP3 vs. BTP4, BTP4 vs. BTP5 using venn and KEGG functional enrichment analysis, ten DEGs involved in anthocyanin biosynthesis pathway were screened. They are F3H (CSA004930), F3′H(CCSA023049), F3′H2(Csng5156), LDOX(CSA011508), CHI(CSA008261), CHS2(CSA026009), CHS2(CSA029707), CHS3(CSA029773), DFR(CSA003949), LAR(CSA018523).

When we compared the expression level of genes involved in the anthocyanin accumulation pertaining to flower coloration, 10, 2 and 21 DEGs in BTP1 vs. BTP2, BTP2 vs. BTP3 and BTP3 vs. BTP4, respectively, were assigned to this pathway, however no DEGs involved in anthocyanin biosynthesis were found in BTP4 vs BTP5. These results suggests that anthocyanin biosynthesis and accumulation mainly occurred in third to fourth stages (BTP3 to BTP4), in particular because 17 structural genes were up-regulated, and only four structural genes downregulated, showing this period to be critical for anthocyanin biosynthesis. Although no new DEG’s were found between BTP4 and BTP5, it is possible that highly-expressed genes involved in anthocyanin synthesis between BTP3 and BTP4 remained expressed between BTP4 and BTP5, and anthocyanins continued to be synthesized despite no new DEGs being found during this stage. This notion is reflected in the heatmap of key structural genes (Figure 7B).

### 2.7. Gene Co-Expression and Crucial Gene Screening Using WGCNA

**Figure 8:**
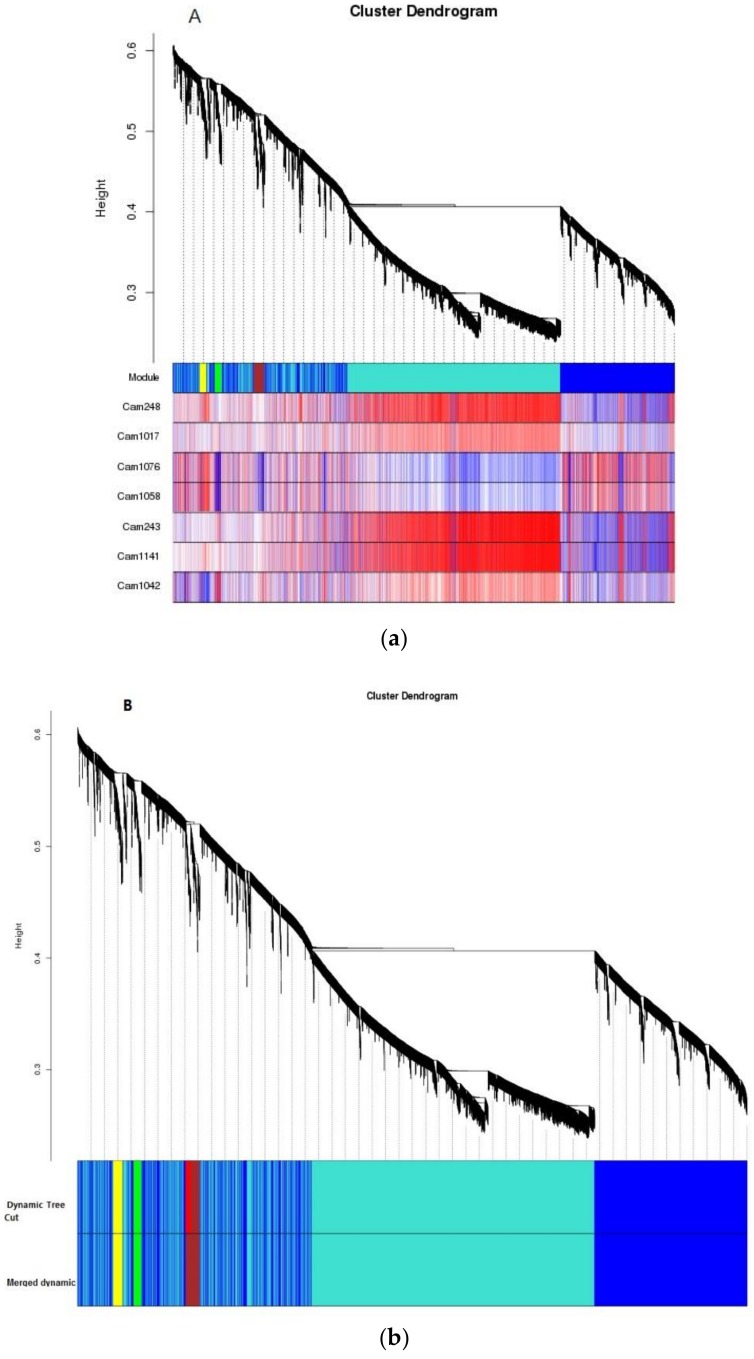

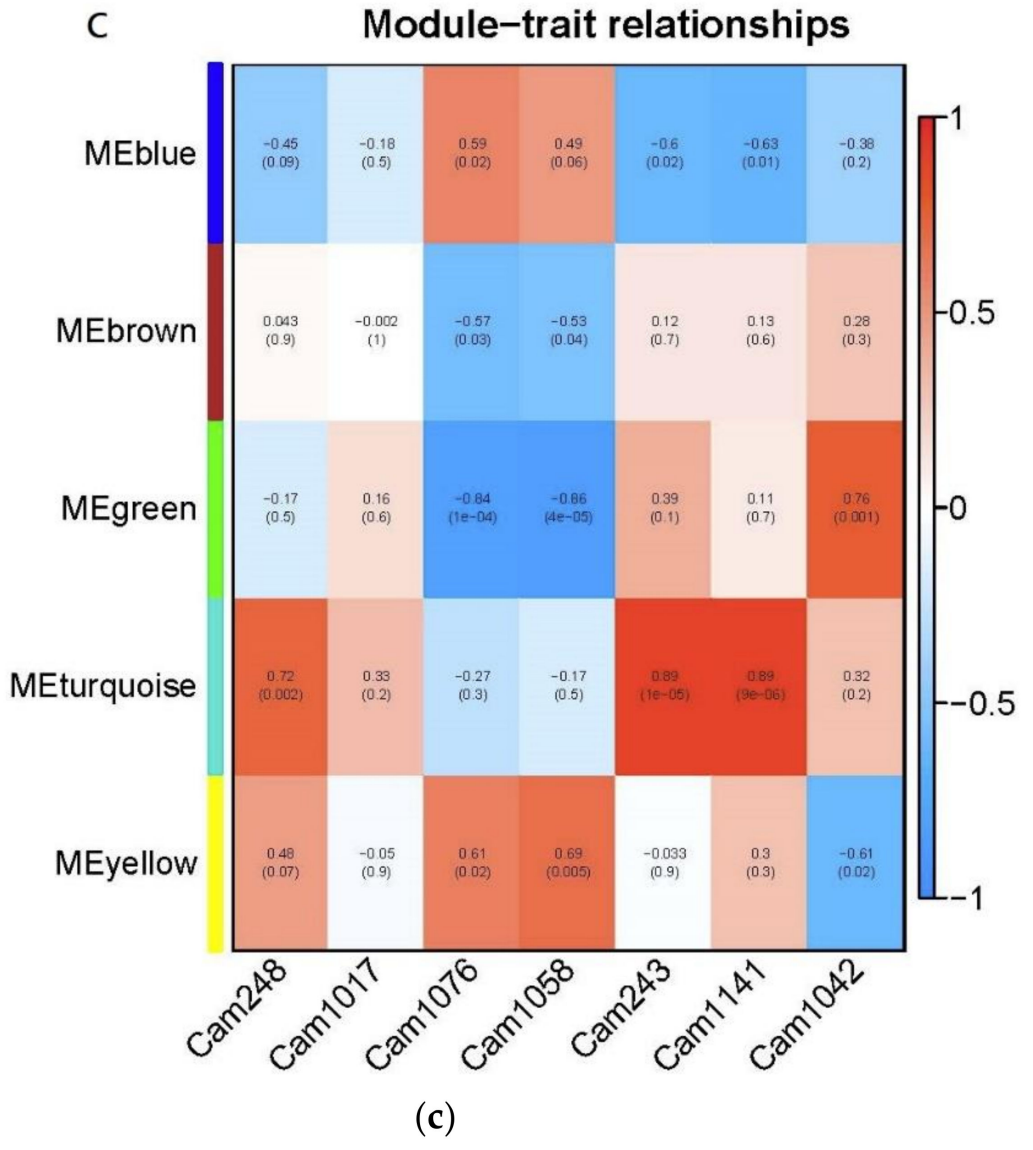
Co-expression and crucial gene screening. (**A**) Clustering results of co-expression modules. Genes in modules are marked with different colors (blue, yellow, brown and turquoise), with grey color representing no genes in any modules. (**B**) Clustering dendrogram. (**C**) Relationships of module and seven different anthocyanins found in BTP flowers.

To reveal the gene expression levels involved in anthocyanins and flavonoid metabolism pathways, modules associated with anthocyanins were obtained by WGCNA. A module can be thought of as a cluster of closely interconnected genes. The interconnectedness of two genes is a combination of adjacency between them, and the strength of connections they share with other “third party” genes. This measure of proximity used by WGCNA is known as topological overlap measure (TOM). Using TOM, WGCNA clusters data into the type of dendrogram “tree” pictured in Figure 8A,B. Individual branches of the tree represent clusters of interconnected genes, which are then defined as “modules.” Each module is measured for co-expression with the trait phenotype, in this case flavonoids, in order to see what cluster of common genes is co-expressed with flavonoids in BTP flowers [37].

Results showed that two modules were significantly correlated with specific anthocyanin and catechin components, as follows: turquoise modules-Cam248 (cyanidin 3-*O*-glucoside), Cam243 (petunidin 3-*O*-glucoside), Cam1141 (epicatechin gallate); green module-Cam1042 (epigallo- catechin); and yellow module-Cam1076 (procyanidin B2), Cam1058 (procyanidin B3). In contrast, the blue module showed a slight relationship with Cam1076 (procyanidin B2), and Cam1058 (procyanidin B3) (Figure 8C). Additionally, procyanidin B2 (cam1076) and procyanidin B3 (cam1058) were significantly negatively correlated with the green module, the correlation coefficients were −0.84 and −0.86, respectively.

The flavonoid-related gene module (turquoise module) was further annotated by GO enrichment analysis. The result showed that dihydroflavonol-4-reductase (DFR, Csng45659), chalcone synthase (CHS, CSA023536, CSA029772, CSA029707, CSA024718, CSA029773), flavanone-dioxygenase-like (F3H-like, CSA004930), chalcone isomerase (CHI, CSA023536), flavonol synthase (FLS, CSA008358) and leucoanthocyanidin dioxygenase isoform 1 (LDOX1, CSA011508) co-expressed (Supplementary Table S7), suggesting expression of these genes have a direct relationship with biosynthesis and accumulation of cyanidin 3-*O*-glucoside, petunidin 3-*O*-glucoside and epicatechin gallate.

### 2.8. Validation of Structural Gene Expression qRT-PCR

**Figure 9:**
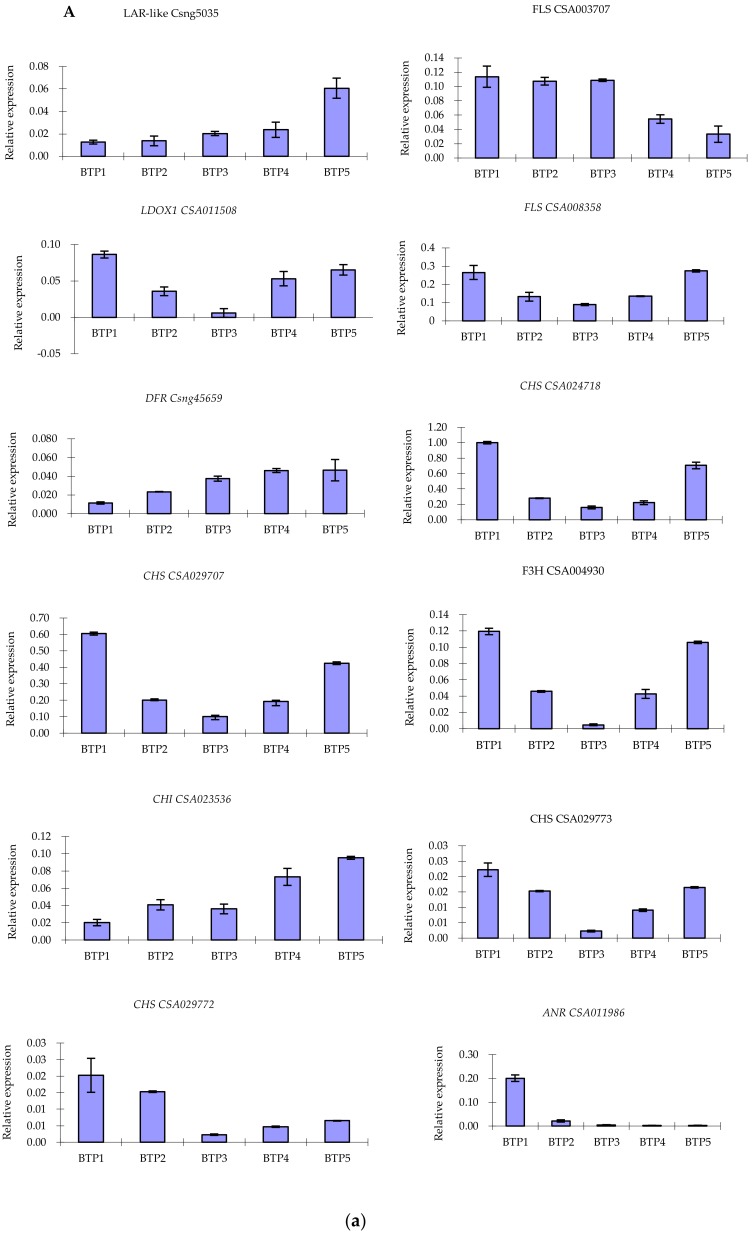

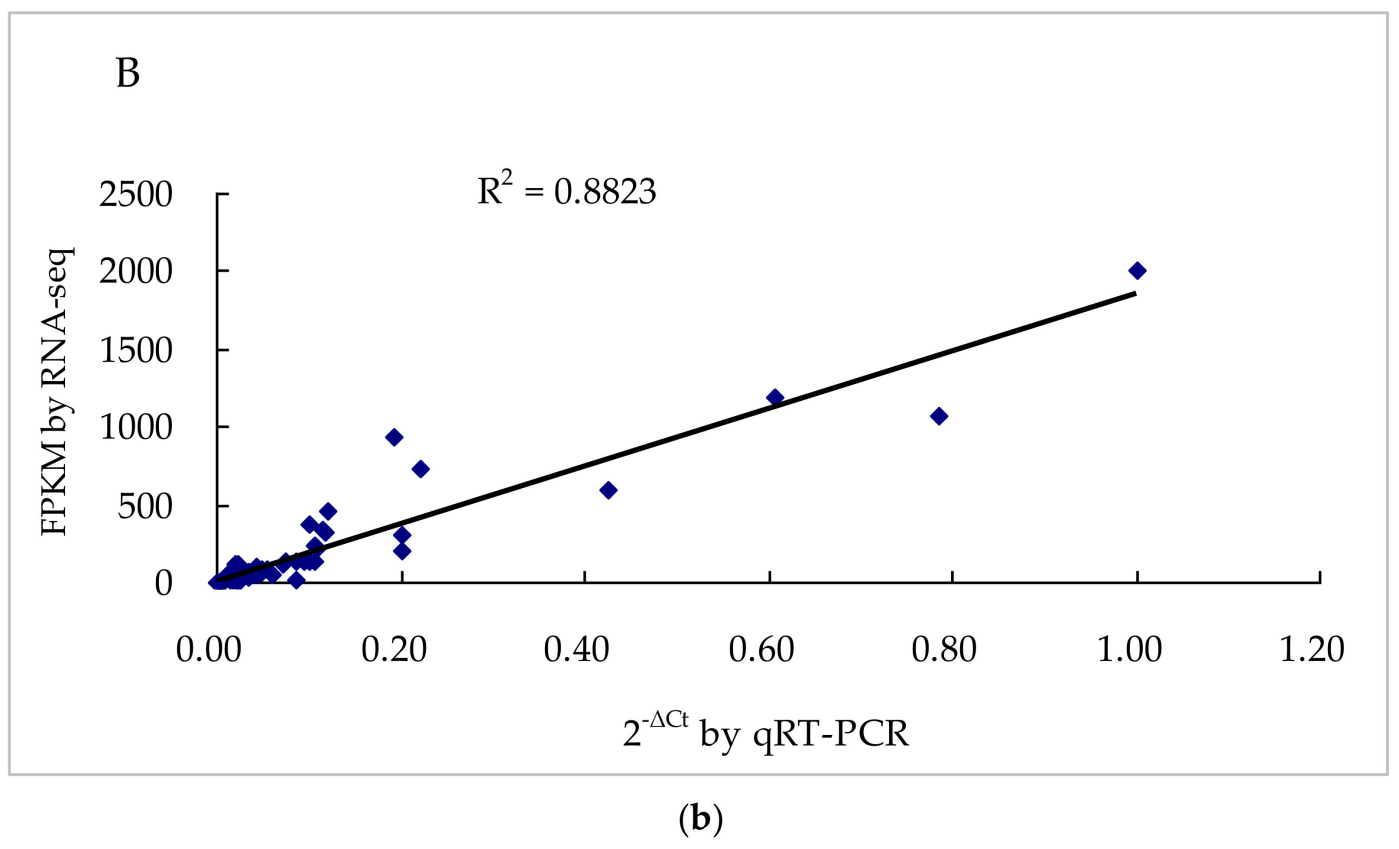
Expression analysis for structural genes involved in anthocyanin accumulation. (**A**) Expression level analysis of key structural genes involved in anthocyanin biosynthesis in pink tea flower development stages. (**B**) Correlation analysis based on RNA-seq and qRT-PCR data.

In order to confirm the accuracy of the high-throughput sequencing results, twelve unigenes involved in anthocyanin metabolic pathway (i.e., CHS, CHI, F3H, FLS, DFR, LDOX, ANR, LAR) were subjected to analysis by qRT-PCR. The results showed that all the twelve unigenes were consistent with RNA-seq datasets. Correlation between FPKM of different unigenes and data of qRT-PCR were determined using the Pearson correlation coefficient. Results showed expression levels between the unigene RNA-seq and qRT-PCR that reflected a significant correlation (correlation coefficient of 0.900, Figure 9). For example, the expression of *LAR-like*(Csng05035), CHI(CSA023536) and DFR(Csng45659) were significantly up-regulated during the tea flower development by qRT-PCR, which was consistent with the expression level (FPKM) from RNA-seq datasets.

## 3. Discussion

Petal color is an important trait that effects the ornamental value of many ornamental plants, and the relationship of color formation with anthocyanin accumulation has been extensively studied for many years [38,39]. Studies indicate that flower color is mainly determined by four classes of natural pigments: chlorophylls, carotenoids, flavonoids, and betalains [40]. Formation of flavonoids, and anthocyanins in particular, have been shown to cause color and color enhancement through increased pigment concentration in petals [41]. Currently, many articles have described key genes and enzymes for flower color formation and flavonoid accumulation in many ornamental flowers, including *Camellia chekiangoleos* [42] and *Camellia nitidissima* [43], although their mechanisms of anthocyanin biosynthesis were not well understood. Prior to this study, the common understanding was that all flower petals of tea (*Camellia sinensis* L.) are white, but the rare pink flowers of BTP have provided unique material to better understand molecular mechanisms behind pigmentation and secondary metabolism in anthocyanin-rich tea plants.

The rapid development of metabolome which reflects the interaction between an organism’s genome and its environment, is widely researched and used. Metabolites can now be identified and quantified using a number of different analysis technologies including NMR spectroscopy and mass spectrometry. Similarly, transcriptome reflects the genes that are being actively expressed at any given time, and transcriptomics are being continually used for investigation and identification of biomarkers in different pathways. As mentioned above, some *Camellia* species have been subject to transcriptome analysis, confirming the involvement of several putative structural genes in petal color formation [20,44,45,46].

In the present study, the techniques of transcriptomics were combined with metabolomics in order to better understand gene expression and flavonoid alterations during BTP flower development. All together 638 metabolic components were identified by UPLC-ESI-MS/MS. To understand metabolic characteristics of pink tea flower development, our attention was focused on anthocyanins and main flavonoid compounds (catechins, kaempferol and quercetin) in all petals samples.

Our results indicated that cyanidin and its glycoside-derived compounds play an important role in color development of pink tea flower. Notably, the levels of three novel anthocyanins, i.e., petunidin 3-*O*-glucoside, cyanidin *O*-syringic acid and pelargonidin 3-*O*-beta-d-glucoside, were significantly increasing during development of pink flowers of BTP. We believe that the accumulation of these novel anthocyanins during flower bloom is causing the unique pink pigmentation of BTP flowers. Although several flavonols, known as branch pathway compounds or metabolic precursor of anthocyanin, such as kaempferol, quercetin, myricetin, afzelechin, were detected, their concentrations showed little change in tea flower development. In all the samples, epigallocatechin (EGC) was the dominant flavonol in pink petals (Table S2). Interestingly, the content of colorless flavonoids among flower samples did not show obvious difference based on flower development, implying that flavonol synthesis was not impacted by the accumulation of anthocyanins.

Flavonoids are made up mainly of anthocyanins, flavones, chalcones, flavanones, flavonols, and isoflavonoids. Of these flavonoid molecules, anthocyanins are widely found to contribute to both flower and fruit colors among flowering plants [47]. And many studies have shown that CHS, CHI, F3′H and F3′5′H in the flavonoid pathway played a major role in flower coloration [47,48].

DFR reduces dihydroflavonols into their subsequent 3,4-cis-leucoanthocyanidins. Some plant species such as grapevine do not synthesize pelargonidin-based anthocyanins, and as a result their DFR cannot utilize dihydrokaempferol (DHK) as a substrate [49]. Due to impact on the metabolic fate of common substrates, the expression patterns of DFR and FLS are regarded as an important determinants of flower color [50]. Flavan-3-ol biosynthesis involves three principal enzymes: LAR, LDOX (also called anthocyanidin synthase, ANS), and ANR. ANR catalyzes the formation of cis-flavan-3-ol from anthocyanidin [51], LAR catalyzes the formation of tran-flavan-3-ol from leucoanthocyanidin [25,52], and LDOX catalyzes the formation of anthocyanidin from flavan- 3,4-diols [53,54]. ANR additionally catalyzes the transformation of anthocyanidin into epicatechin [51], and can catalyze the transformation of an anthocyanidin into an anthocyanin [55].

In the present study, several critical genes were highly expressed consistently throughout tea flower development, and certain genes exhibited behavior that varied along with flower development. For example, almost all dominant genes, such as CHS-like (CSA026009), and FLS (CSA003707) showed expression levels slightly down-regulated during the tea floral development, which would enhance anthocyanin biosynthesis in flowers due to the fact that FLS competes for substrate with anthocyanin biosynthesis gene, DFR. Functioning along slightly different lines, secondary dominant genes, such as F3’H(CSA023049), CHS (CSA024718), and CHS3 (CSA029707), showed expression levels down-regulated from the first to second stage, and slightly up-regulated from the third through the fifth stage. By using Venn and KEGG functional enrichment analysis during flower development, ten DEGs involved in anthocyanin biosynthesis pathway were screened. The analyses showed that anthocyanin biosynthesis and accumulation mainly occurred between the third to fourth stages (BTP3 to BTP4). Notably, we discovered three novel anthocyanins in the pink flowers of BTP (cyanidin *O*-syringic acid, pelargonidin 3-*O*-beta-d-glucoside and petunidin 3-*O*-glucoside), which correlated significantly with pink flower development and may explain the unique pigmentation of this tea flower. A deep understanding of the anthocyanin phenotypes and biosynthetic mechanisms of the pink flowers of the BTP purple-leaf cultivar can provide useful information for future research on topics such as ornamental *Camellia* flower pigmentation, the function of flavonoids in plant reproductive tissue, and the relationship between flavonoid biosynthesis in vegetative tissue and that in reproductive tissue.

## 4. Materials and Methods

### 4.1. Plant Materials

Flower samples were collected from three-year-old purple-leaf cultivar tea plants (*Camellia sinensis* L.), cultivated on a tea plantation in Boluo, Guangdong Province, China (23°97′ N, 114°28′ E) under natural conditions. A new purple-leaf cultivar, Baitang purple tea (BTP) was discovered as a natural anthocyanin-rich mutant in the Baitang Mountains of Guangdong. As with all anthocyanin-rich tea varieties, BTP’s stem, bud, and young two to three leaves are purple, and then fade to green as leaves increase in maturity lower down the stalk. The most significant feature of BTP is that it has a pink flower in winter. Flower samples at five stages of development were collected from BTP flower cultivar on the same day, 13 November 2017. The five stages of development in the flower samples gathered were: Flower bud stage (BTP1), petals beginning to split stage (BTP2), half bloom stage (BTP3), full bloom stage (BTP4) and wither stage (BTP5). After careful selection and removal from the tea plant, samples were immediately frozen in liquid nitrogen and stored in -80℃freezers for metabolite and RNA sequences analysis. For all flower samples, three biological replicates from different tea trees were collected at each of the five stages of growth, with each biological replicate containing 10 floral buds.

### 4.2. Samples Extraction and Targeted Metabolomics Analysis

#### 4.2.1. Extraction of Secondary Metabolites

The petal samples frozen with liquid N were ground into powder, and then 100 mg of each powder sample was inserted into 1.5 mL EP tubes. Extraction was performed using 1.0 mL 70% aqueous methanol for 24 h at 4 °C. Following a 10 min centrifugation (10,000× *g*) at 4 °C. The extracts were then filtrated by using 0.22 μm pore size nylon membrane before LC-MS analysis. All quality-control (QC) samples were prepared by extracting and mixing the three replicate samples taken from each flower type at each stage of floral development. During the instrumental analysis, each QC sample was measured alongside its corresponding set of three experimental samples in order to examine the stability of the analytical conditions.

#### 4.2.2. Liquid Chromatographic Mass Spectrometry Analysis

The various flower extracts (10 μL) were subject to metabolic profiling analysis using UPLC-ESI-MS/MS system (Shimadzu, Kyoto, Japan). The chromatographic separations were conducted on a Waters Acquity UPLC HSS T3 C18 column (2.1 mm × 100 mm, i.d., 1.8 µm) (Waters Corp., Milford, MA, USA) at 40 °C. The mobile phase consisted of water with 0.04% acetic acid (mobile phase A) and acetonitrile with 0.04% acetic acid (mobile phase B). A Linear gradient program for elution was set as follows: from 5% to 95% B for 0–11.0 min, from 95% to 5% for 11.0–12.0 min, from 5% to 5% for 12.1–15.0 min. The flow rate of mobile phase was 0.40 mL/min.

#### 4.2.3. Metabolite Identification and Quantification

The MS acquisition and MS/MS analysis were performed using mass spectrometer system (API 4500 Q TRAP LC/MS/MS System, AB SCIEX, Framingham, MA, USA). The electro-spray ionization source (ESI) source method involved the following parameters: turbo spray, ion source; source temperature of 550 °C; ion spray voltage of 5.5 kV; air curtain gas pressure 25 pounds per square inch (psi), ion source gas 1 pressure of 55 psi, and gas II pressure 60 psi. Multiple reaction monitoring (MRM) experiments using 5 psi nitrogen collision gas were performed to acquire QQQ scans.

Metabolite identification was based on the metabolite information public database, in addition to the MetWare database of Metware Biotechnology Company (Wuhan, China). Supplementary mass spectrometry databases were referenced in order to conduct metabolite qualitative analysis of MS data. These open database sources included HMDB, MoToDB, MassBank, METLIN, and KNAPSAcK. Structural analysis of metabolites was determined using standard metabolic operating procedures. MRM was used to conduct metabolite quantification. All metabolites identified were subject to partial least squares discriminant analysis. Principle component analysis (PCA) and Orthogonal Partial Least Squares Discriminate Analysis (OPLS-DA) were carried out to identify potential biomarker variables. For potential biomarker selection, variable importance in projection (VIP) ≥ 1 and fold change (FC) ≥ 2 or ≤ 0.5 were set for metabolites with significant differences.

### 4.3. RNA Extraction, and Illumina Sequencing

Total RNA from each frozen sample was extracted and isolated using modified CTAB method. RNA samples were assessed for contamination and degradation using agarose gel electrophoresis. The concentration of RNA was measured using NanoDrop 2000 spectrophotometer (Thermo Fisher Scientific, Waltham, MA, USA). The integrity of RNA was assessed using Agilent 2100 system (Agilent Technologies Inc., Palo Alto, CA, USA). The RNA-seq was performed on three replicates for every sample used. The RNA-seq assembly and analysis was conducted by Beijing Biomarker Biotechnology Corporation (Beijing, China) and Beijing Biomarker Cloud technology Corporation (Beijing, China). The RNA sequencing libraries were generated using NEBNext® Ultra™ II RNA Library Prep Kit for Illumina® (New England Biolabs Inc., Ipswich, MA, USA) following the protocols and sequences attributed to each sample by adding index codes. The libraries were sequenced on Illumina® HiSeq2500 platform (Illumina Inc., San Diego, CA, USA) following the manufacturer’s protocols. The raw sequence reads were filtered by removing low quality reads and adaptors, and raw sequences were changed into clean reads. The remaining clean reads were mapped to the reference genome (http://www.plantkingdomgdb.com/tea_tree/). The Hierarchical Indexing for Spliced Alignment of Transcripts (HISAT 2) program was used to map to the reference genome [56].

### 4.4. Gene Functional Annotation and Expression Analysis

Gene function was annotated according to these databases: (NCBI non-redundantprotein sequences(Nr); Clusters of Orthologous Groups of proteins (COG/KOG); Swiss PROT protein sequence database; Kyoto Encyclopedia of Genes and Genomes (KEGG,); homologous protein family(Pfam) and Gene Ontology(GO). The software DESeq was used to conduct differential expression analysis among samples [57]. The Benjamini-Hochberg approach was used to determine a significant *p*-value. DEGs were screened by DESeq software when adjusted *p*-value was less than 0.05. GO analysis of the differentially expressed genes (DEGs) was conducted using the GOseq R package software [58]. Identification of enriched pathways of DEGs was tested using KEGG Orthology Based Annotation System (KOBAS) software [59]. The unigene expression levels were quantified using the values of fragments per kilobase of transcript per million fragments mapped [60].

### 4.5. Gene screening by Co-Expression Analysis

Weighted correlation network analysis, also known as weighted gene co-expression network analysis (WGCNA), is a widely used data mining technique for researching networks of biological metabolism and regulation based on evaluation correlations between variables [61]. WGCNA can be used to construct global co-expression networks and identify clusters of interconnected genes [62]. WGCNA can be conducted using correlation networks, which eventually facilitate the identification of candidate biomarkers involved in biological processes [63,64]. In this study, WGCNA was applied to construct a tea flower gene co-expression network, and the modules of highly correlated genes were identified by normalized expression matrix data. According to Topological Overlap Matrix measure [65], unigenes were clustered first hierarchically. Eventually, the co-expression models were identified using a bottom-up algorithm, known as the dynamic hybrid cut method. The gene significance (GS), a measure of correlation between genes and sample traits, was calculated for each module in order to find connectivity between genes and sample traits [66]. Five important anthocyanin and two flavan-3-ol components were defined as sample traits; Cam248-cyanidin 3-*O*-glucoside; Cam1017-cyanidin *O*-syringic acid; Cam1076-procyanidin B2; Cam1058-procyanidin B3; Cam243-petunidin 3-*O*-glucoside; Cam1141-eEpicatechin gallate; Cam1042-epigallocatechin.

### 4.6. Gene Validation and Expression Analysis

Twelve unigenes related to anthocyanin biosythesis in the pink tea flower were selected for validation using quantitative real-time PCR (qRT-PCR). The specific qRT-PCR primers were designed (Supplementary Table S6). The qRT-PCR was conducted using the LightCycler® 480 II Real-Time System (LightCycler® 480 II cycler, Roche, Carlsbad, CA, USA) with a 96-well plate. The thermal profile for the PCR amplification was 95 °C for 5 min, followed by 40 cycles of 10 s at 95 °C and another 40 cycles at 60 °C for 30 s. The HieffTM qPCR SYBR Green Master Mix (No Rox) (Yeasen Biotech Co., Ltd., Shanghai, China) was used for all PCR reactions according to the instruction’s protocol. All qRT-RCRs analyses were conducted using three technical and three biological replicates. The reference gene (β-actin gene) was used as an internal expression control. The expression level of different genes to the control was calculated according to the 2−ΔΔCT method [67].

### 4.7. Statistical Analysis

All the statistical analyses were conducted using ANOVAB with SPSS V20.0 (SPSS Inc., Chicago, IL, USA). Some charts were prepared using Excel 2010. The content of anthocyanin was expressed with Mean ± SD (standard error).

## 5. Conclusions

In this study, the molecular mechanisms driving anthocyanin accumulation in the development of mutant pink tea flowers (*Camellia sinensis* L.) were analyzed. At least 12 anthocyanins were detected in petals, and among these metabolites, three anthocyanins not previously detected in *Camellia sinensis* L. (cyanidin *O*-syringic acid, petunidin 3-*O*-glucoside, and pelargonidin 3-*O*-beta-d-glucoside) were found, and correlated with pink flower development, perhaps explaining the unique pigmentation of this tea flower. 21 differentially expressed genes (DEGs) involved in anthocyanin pathway were identified using KEGG pathway functional enrichment, and 10 key structural genes involved in anthocyanin accumulation during flower development were identified using Venn and KEGG analysis. Weighted gene co-expression analysis (WGCNA) was used to identify 10 critical genes that had correlated with high levels of biosythesis and accumulation of cyanidin 3-*O*-glucoside, petunidin 3-*O*-glucoside and epicatechin gallate. Anthocyanin biosynthesis and accumulation mainly occurred between the third and fourth flower development stages. Our results revealed the special expression patterns of the critical anthocyanin genes coinciding with pink tea flower development. These results provide useful information about the molecular mechanisms of coloration in the rare pink tea flower of anthocyanin-rich BTP tea. This study will be useful for the further research on anthocyanin accumulation in purple tea, pigmentation in ornamental camellia flowers, and the relationship of flavonoid biosynthesis in vegetative and reproductive plant tissue.

## Supplementary Materials

Supplementary Materials are available online.
