# Supplementary material for: Metabolome and Transcriptome Sequencing Analysis Reveals Anthocyanin Metabolism in Pink Flowers of Anthocyanin-Rich Tea (Camellia sinensis)
Source: Molecules. 2019 Mar 18;24(6):1064. doi: 10.3390/molecules24061064 (PMC6471635; doi:10.3390/molecules24061064)
Supplement: Supplementary file 1 [file molecules-24-01064-s001.pdf]

Supplementary Table 1 type and content of anthocyanins in different tea floral development stages

Index	Molecular weight(Da)	Compounds	Cps : count per second									
			BTP1	SE	BTP2	SE	BTP3	SE	BTP4	SE	BTP5	SE
Cam248	Cyanidin 3-O-glucoside*	449.10	7.04E+07	5.01E+06	5.54E+07	5.85E+06	5.55E+07	8.66E+06	4.33E+07	5.74E+06	5.48E+07	9.47E+06
Cam463	Peonidin	301.10	2.43E+04	8.66E+03	1.74E+04	1.27E+04	1.64E+04	9.47E+03	1.46E+04	5.74E+03	2.37E+04	1.59E+04
Cam1026	Luteolin O-hexosyl-O-hexosyl-O-hexoside	772.10	1.54E+04	7.32E+03	1.98E+04	6.21E+03	3.11E+04	8.75E+03	2.50E+04	2.63E+03	2.91E+04	5.59E+03
Cam1017	Cyanidin O-syringic acid*	466.10	1.60E+07	1.81E+06	1.29E+07	1.16E+06	1.37E+07	2.79E+06	1.05E+07	3.85E+06	1.53E+07	2.10E+06
Cam1076	Procyanidin B2	578.14	3.73E+07	8.26E+06	3.62E+07	6.37E+06	3.73E+07	8.65E+06	4.08E+07	3.07E+06	4.53E+07	3.53E+06
Cam1058	Procyanidin B3	578.14	3.98E+07	9.33E+06	3.87E+07	6.69E+06	3.86E+07	8.06E+06	4.21E+07	3.40E+06	4.54E+07	2.64E+06
Cam272	Cyanidin 3-O-rutinoside	595.00	7.61E+06	4.59E+06	6.31E+06	3.53E+06	6.07E+06	3.59E+06	6.15E+06	2.23E+06	6.64E+06	1.39E+06
Cam222	Cyanidin 3,5-O-diglucoside*	611.00	1.54E+07	4.92E+06	1.21E+07	4.84E+06	1.16E+07	3.16E+06	1.03E+07	4.04E+06	9.06E+06	1.73E+06
Cam249	Malvidin 3,5-diglucoside	655.20	2.62E+04	1.16E+04	2.21E+04	7.04E+03	2.36E+04	7.97E+03	1.86E+04	8.21E+03	2.06E+04	5.60E+03
Cam250	Pelargonin	595.00	5.41E+05	2.99E+05	4.35E+05	1.62E+05	4.13E+05	7.31E+04	3.88E+05	2.06E+05	2.36E+05	5.64E+04
Cam243	Petunidin 3-O-glucoside*	479.00	5.46E+07	1.38E+07	2.55E+07	3.33E+06	2.23E+07	5.05E+06	1.41E+07	5.33E+06	1.51E+07	4.84E+06
Cam266	Pelargonidin 3-O-beta-D-glucoside	433.10	2.33E+06	1.10E+06	1.52E+06	5.74E+05	1.33E+06	2.41E+05	1.04E+06	5.08E+05	1.44E+06	3.79E+05

Blue font indicates anthocyanins compounds ~~found~~ discovered in tea flowers for the first time. An ~~;the~~ asterisk in the second column ~~column means~~ indicates predominant products of ~~that main components~~ the ~~involved in~~ anthocyanin biosynthetic pathway in BTP flowers (excluding procyanidins)~~sis~~.

Formatted: Font: Italic

Supplementary Table 2 type and content of main flavonoids compounds in different tea floral development stages

Formatted: Font: Italic

Index	Compounds	Molecular weight(Da)	Cps ： count per second									
			BTP1	SE	BTP2	SE	BTP3	SE	BTP4	SE	BTP5	SE
Cam1220	Dihydrokaempferol	288.063	7.50E+05	6.82E+04	7.42E+05	2.41E+05	7.20E+05	4.80E+05	6.51E+05	4.34E+05	8.31E+05	6.61E+05
Cam1176	Dihydroquercetin	304.058	1.89E+06	1.73E+05	8.19E+05	1.76E+05	4.45E+05	2.71E+04	5.08E+05	1.60E+05	9.04E+05	5.33E+05
Cam1099	Dihydromyricetin	320.053	6.37E+06	1.11E+06	4.30E+06	3.75E+05	3.66E+06	1.26E+06	2.97E+06	1.83E+05	3.23E+06	8.06E+05
Cam629	Kaempferol	286.048	8.14E+06	4.55E+06	6.66E+06	3.07E+06	5.42E+06	1.85E+06	4.39E+06	1.98E+06	5.47E+06	1.59E+06
Cam601	Quercetin	302.043	1.77E+06	5.89E+05	1.82E+06	8.03E+05	1.49E+06	5.17E+05	1.09E+06	4.27E+05	1.04E+06	2.57E+05
Cam547	Myricetin	318.038	2.41E+05	1.42E+05	1.88E+05	1.19E+05	1.24E+05	1.07E+05	9.01E+04	5.14E+04	9.93E+04	9.08E+04
Cam1095	Afzelechin	274.084	1.42E+05	6.36E+04	1.82E+05	9.29E+04	1.75E+05	7.66E+04	1.66E+05	6.96E+04	1.92E+05	9.68E+04
Cam1054	Catechin	290.079	3.20E+07	1.64E+06	3.10E+07	1.53E+06	2.86E+07	1.46E+06	2.59E+07	2.34E+06	2.43E+07	1.46E+06
Cam1015	Gallocatechin	306.074	5.94E+07	3.19E+06	5.25E+07	2.75E+06	5.06E+07	4.23E+06	4.45E+07	1.86E+06	4.11E+07	2.57E+06
Cam1116	Epicatechin-epiafzelechin	562.1	5.15E+04	3.63E+04	5.79E+04	1.91E+04	5.93E+04	1.58E+04	6.37E+04	2.10E+04	7.36E+04	4.88E+04
Cam346	L-Epicatechin	290.3	3.71E+07	1.93E+06	3.65E+07	9.50E+05	3.67E+07	1.11E+06	3.48E+07	1.40E+06	3.64E+07	1.57E+06
Cam1141	Epicatechin gallate	442.3	1.40E+08	8.14E+06	1.27E+08	5.29E+06	1.22E+08	2.52E+06	1.16E+08	4.04E+06	1.17E+08	8.66E+06
Cam1042	Epigallocatechin	306	8.16E+07	1.22E+07	8.04E+07	6.80E+06	7.96E+07	6.92E+06	7.49E+07	5.46E+06	7.74E+07	2.26E+06
Cam348	Epigallate catechin gallate	458.085	4.01E+07	2.72E+06	3.30E+07	1.48E+06	3.07E+07	1.83E+06	2.84E+07	1.87E+06	2.75E+07	1.95E+06
Cam1042	Epigallocatechin	306	8.16E+07	1.22E+07	8.04E+07	6.80E+06	7.96E+07	6.92E+06	7.49E+07	5.46E+06	7.74E+07	2.26E+06
Cam1120	Gallocatechin-catechin	594.1	9.23E+06	2.13E+06	1.13E+07	3.07E+06	1.23E+07	2.79E+06	1.09E+07	2.48E+06	9.98E+06	3.01E+06

Supplementary Table 3. The quality of RNA-seq in pink tea flower

**Samples Name**	Clean reads	Clean bases	GC Content	%≥Q30
BTP11	24,401,173	7,272,491,122	44.91%	85.34%
BTP12	21,739,300	6,470,253,566	44.38%	85.88%
BTP13	21,682,289	6,448,733,710	44.57%	85.29%
BTP21	20,915,131	6,229,932,294	45.22%	85.24%
BTP22	22,420,616	6,674,212,962	44.78%	85.01%
BTP23	21,883,981	6,513,593,344	44.73%	85.21%
BTP31	20,935,452	6,220,494,474	45.17%	85.01%
BTP32	21,362,041	6,362,838,760	45.30%	85.73%
BTP33	23,929,435	7,138,488,744	44.90%	85.05%
BTP41	25,751,867	7,692,097,820	45.21%	87.27%

BTP42	24,301,648	7,234,121,756	45.28%	88.20%
BTP43	24,896,357	7,406,220,944	44.88%	88.16%
BTP51	21,291,989	6,355,399,448	45.26%	87.70%
BTP52	22,152,462	6,614,402,460	45.24%	87.49%
BTP53	28,163,774	8,376,422,666	45.03%	88.19%

1–3: Three biological replicates of each tissue at tea flower development stage; Raw reads: original number of reads obtained by sequencing; Clean reads: number of reads after removing low-quality reads and trimming adapter sequences; Clean bases: number of clean reads multiplied by length of clean reads. Q30: Phred score, indicates 99% and 99.9% accuracy of sequenced bases; GC content: percentage of G and C in total bases.

Supplementary Table 4. Clean reads mapped to the reference genome in tea flower development.

Samples Name	Total Reads	Mapped Reads	Uniq Mapped Reads	Multiple Map Reads	Reads Map to '+'	Reads Map to '-'
BTP11	48,802,346	37,293,853 (76.42%)	36,008,165 (73.78%)	1,285,688 (2.63%)	18,493,535 (37.89%)	18,610,192 (38.13%)
BTP12	43,478,600	32,962,390 (75.81%)	31,853,261 (73.26%)	1,109,129 (2.55%)	16,347,844 (37.60%)	16,433,698 (37.80%)

BTP13	43,364,578	33,092,335 (76.31%)	32,013,598 (73.82%)	1,078,737 (2.49%)	16,402,973 (37.83%)	16,503,569 (38.06%)
BTP21	41,830,262	31,589,683 (75.52%)	30,454,831 (72.81%)	1,134,852 (2.71%)	15,652,381 (37.42%)	15,767,875 (37.69%)
BTP22	44,841,232	34,037,671 (75.91%)	32,892,099 (73.35%)	1,145,572 (2.55%)	16,870,426 (37.62%)	16,987,705 (37.88%)
BTP23	43,767,962	33,392,416 (76.29%)	32,315,261 (73.83%)	1,077,155 (2.46%)	16,553,103 (37.82%)	16,670,821 (38.09%)
BTP31	41,870,904	31,933,307 (76.27%)	30,830,111 (73.63%)	1,103,196 (2.63%)	15,834,315 (37.82%)	15,933,041 (38.05%)
BTP32	42,724,082	32,989,600 (77.22%)	31,856,435 (74.56%)	1,133,165 (2.65%)	16,352,938 (38.28%)	16,465,577 (38.54%)
BTP33	47,858,870	36,838,098 (76.97%)	35,628,007 (74.44%)	1,210,091 (2.53%)	18,256,902 (38.15%)	18,375,347 (38.39%)
BTP41	51,503,734	39,406,493 (76.51%)	37,950,544 (73.69%)	1,455,949 (2.83%)	19,538,002 (37.94%)	19,622,512 (38.10%)
BTP42	48,603,296	36,630,157 (75.37%)	35,339,355 (72.71%)	1,290,802 (2.66%)	18,164,886 (37.37%)	18,245,513 (37.54%)
BTP43	49,792,714	38,744,562 (77.81%)	37,392,013 (75.10%)	1,352,549 (2.72%)	19,211,077 (38.58%)	19,273,247 (38.71%)
BTP51	42,583,978	32,400,843 (76.09%)	31,230,927 (73.34%)	1,169,916 (2.75%)	16,064,823 (37.73%)	16,136,608 (37.89%)
BTP52	44,304,924	33,625,913 (75.90%)	32,446,166 (73.23%)	1,179,747 (2.66%)	16,688,854 (37.67%)	16,752,348 (37.81%)
BTP53	56,327,548	43,193,996 (76.68%)	41,523,939 (73.72%)	1,670,057 (2.96%)	21,409,569 (38.01%)	21,504,543 (38.18%)

Total	691,655,030	528,131,317(76.34%)	509,734,712(73.68%)	1,8396,605(2.65%)	261,841,628(37.85%）	263,282,596（38.06%）

GC content: Clean Data GC content，the percentage of G and C base number in the total base number; ≥Q30: The percentage of base number which qualities of Clean Data is greater than or equal to 30 in the total base number, the number of Clean Reads is single ended calculation; Mapped Reads: The number of Reads which are compared to the reference genome and the percentage of this in Clean Reads; Uniq Mapped Reads: The number of Reads which are compared to the reference genome in single location and the percentage of this in Clean Reads.

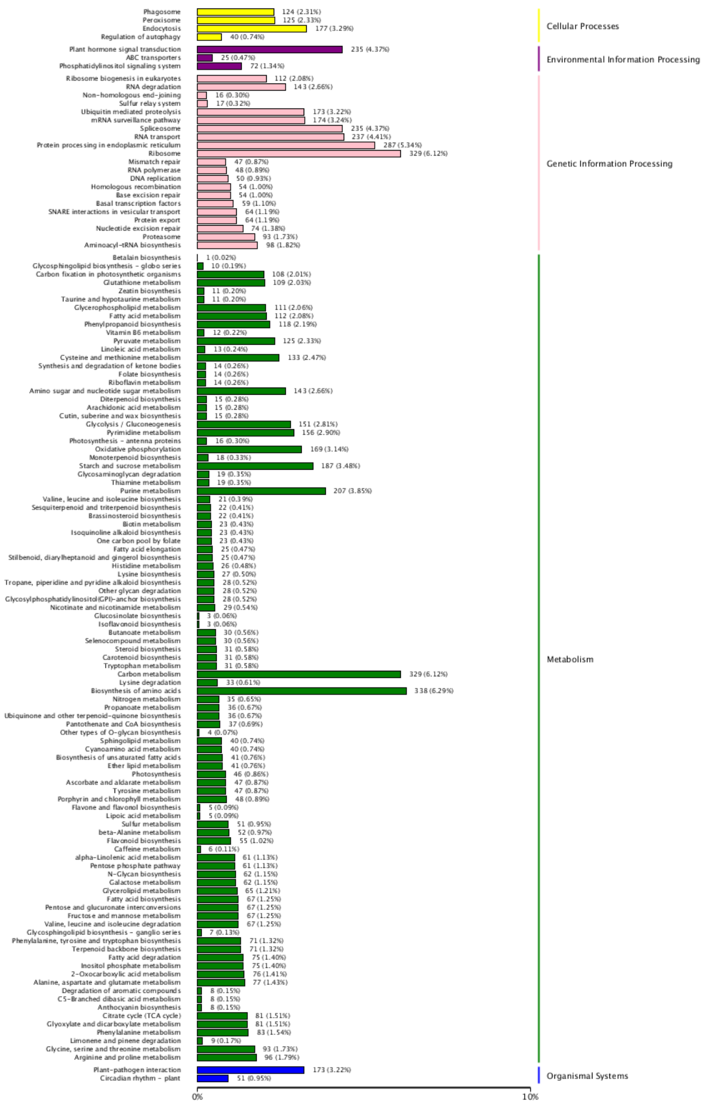
Phagosome 124 (2.31%)
Peroxisome 125 (2.33%)
Endocytosis 177 (3.29%)
Regulation of autophagy 40 (0.74%)
Plant hormone signal transduction 235 (4.37%)
ABC transporters 25 (0.47%)
Phosphatidylinositol signaling system 72 (1.34%)
Ribosome biogenesis in eukaryotes 112 (2.08%)
RNA degradation 143 (2.66%)
Non-homologous end-joining 16 (0.30%)
Sulfur relay system 17 (0.32%)
Ubiquitin mediated proteolysis 173 (3.22%)
mRNA surveillance pathway 174 (3.24%)
Spliceosome 235 (4.37%)
RNA transport 237 (4.41%)
Protein processing in endoplasmic reticulum 287 (5.34%)
Ribosome 329 (6.12%)
Mismatch repair 47 (0.87%)
RNA polymerase 48 (0.89%)
DNA replication 50 (0.93%)
Homologous recombination 54 (1.00%)
Base excision repair 54 (1.00%)
Basal transcription factors 59 (1.10%)
SNARE interactions in vesicular transport 64 (1.19%)
Protein export 64 (1.19%)
Nucleotide excision repair 74 (1.38%)
Proteasome 93 (1.73%)
Aminoacyl-tRNA biosynthesis 98 (1.82%)
Betalain biosynthesis 1 (0.02%)
Glycosphingolipid biosynthesis - globo series 10 (0.19%)
Carbon fixation in photosynthetic organisms 108 (2.01%)
Glutathione metabolism 109 (2.03%)
Zeatin biosynthesis 11 (0.20%)
Taurine and hypotaurine metabolism 11 (0.20%)
Glycerophospholipid metabolism 111 (2.06%)
Fatty acid metabolism 112 (2.08%)
Phenylpropanoid biosynthesis 118 (2.19%)
Vitamin B6 metabolism 12 (0.22%)
Pyruvate metabolism 125 (2.33%)
Linoleic acid metabolism 13 (0.24%)
Cysteine and methionine metabolism 133 (2.47%)
Synthesis and degradation of ketone bodies 14 (0.26%)
Folate biosynthesis 14 (0.26%)
Riboflavin metabolism 14 (0.26%)
Amino sugar and nucleotide sugar metabolism 143 (2.66%)
Diterpenoid biosynthesis 15 (0.28%)
Arachidonic acid metabolism 15 (0.28%)
Cutin, suberine and wax biosynthesis 15 (0.28%)
Glycolysis / Gluconeogenesis 151 (2.81%)
Pyrimidine metabolism 156 (2.90%)
Photosynthesis - antenna proteins 16 (0.30%)
Oxidative phosphorylation 169 (3.14%)
Monoterpenoid biosynthesis 18 (0.33%)
Starch and sucrose metabolism 187 (3.48%)
Glycosaminoglycan degradation 19 (0.35%)
Thiamine metabolism 19 (0.35%)
Purine metabolism 207 (3.85%)
Valine, leucine and isoleucine biosynthesis 21 (0.39%)
Sesquiterpenoid and triterpenoid biosynthesis 22 (0.41%)
Brassinosteroid biosynthesis 22 (0.41%)
Biotin metabolism 23 (0.43%)
Isoquinoline alkaloid biosynthesis 23 (0.43%)
One carbon pool by folate 23 (0.43%)
Fatty acid elongation 25 (0.47%)
Stilbenoid, diarylheptanoid and gingerol biosynthesis 25 (0.47%)
Histidine metabolism 26 (0.48%)
Lysine biosynthesis 27 (0.50%)
Tropane, piperidine and pyridine alkaloid biosynthesis 28 (0.52%)
Other glycan degradation 28 (0.52%)
Glycosylphosphatidylinositol(GPI)-anchor biosynthesis 28 (0.52%)
Nicotinate and nicotinamide metabolism 29 (0.54%)
Glucosinolate biosynthesis 3 (0.06%)
Isoflavonoid biosynthesis 3 (0.06%)
Butanoate metabolism 30 (0.56%)
Selenocompound metabolism 30 (0.56%)
Steroid biosynthesis 31 (0.58%)
Carotenoid biosynthesis 31 (0.58%)
Tryptophan metabolism 31 (0.58%)
Carbon metabolism 329 (6.12%)
Lysine degradation 33 (0.61%)
Biosynthesis of amino acids 338 (6.29%)
Nitrogen metabolism 35 (0.65%)
Propanoate metabolism 36 (0.67%)
Ubiquinone and other terpenoid-quinone biosynthesis 36 (0.67%)
Pantothenate and CoA biosynthesis 37 (0.69%)
Other types of O-glycan biosynthesis 4 (0.07%)
Sphingolipid metabolism 40 (0.74%)
Cyanoamino acid metabolism 40 (0.74%)
Biosynthesis of unsaturated fatty acids 41 (0.76%)
Ether lipid metabolism 41 (0.76%)
Photosynthesis 46 (0.86%)
Ascorbate and aldarate metabolism 47 (0.87%)
Tyrosine metabolism 47 (0.87%)
Porphyrin and chlorophyll metabolism 48 (0.89%)
Flavone and flavonol biosynthesis 5 (0.09%)
Lipoic acid metabolism 5 (0.09%)
Sulfur metabolism 51 (0.95%)
beta-Alanine metabolism 52 (0.97%)
Flavonoid biosynthesis 55 (1.02%)
Caffeine metabolism 6 (0.11%)
alpha-Linolenic acid metabolism 61 (1.13%)
Pentose phosphate pathway 61 (1.13%)
N-Glycan biosynthesis 62 (1.15%)
Galactose metabolism 62 (1.15%)
Glycerolipid metabolism 65 (1.21%)
Fatty acid biosynthesis 67 (1.25%)
Pentose and glucuronate interconversions 67 (1.25%)
Fructose and mannose metabolism 67 (1.25%)
Valine, leucine and isoleucine degradation 67 (1.25%)
Glycosphingolipid biosynthesis - ganglio series 7 (0.13%)
Phenylalanine, tyrosine and tryptophan biosynthesis 71 (1.32%)
Terpenoid backbone biosynthesis 71 (1.32%)
Fatty acid degradation 75 (1.40%)
Inositol phosphate metabolism 75 (1.40%)
2-Oxocarboxylic acid metabolism 76 (1.41%)
Alanine, aspartate and glutamate metabolism 77 (1.43%)
Degradation of aromatic compounds 8 (0.15%)
C5-Branched dibasic acid metabolism 8 (0.15%)
Anthocyanin biosynthesis 8 (0.15%)
Citrate cycle (TCA cycle) 81 (1.51%)
Glyoxylate and dicarboxylate metabolism 81 (1.51%)
Phenylalanine metabolism 83 (1.54%)
Limonene and pinene degradation 9 (0.17%)
Glycine, serine and threonine metabolism 93 (1.73%)
Arginine and proline metabolism 96 (1.79%)
Plant-pathogen interaction 173 (3.22%)
Circadian rhythm - plant 51 (0.95%)
0%
10%
Cellular Processes
Environmental Information Processing
Genetic Information Processing
Metabolism
Organismal Systems

Supplementary Figure S1. KEGG classification of assembled unigenes. The 6,041 KO annotated unigenes were assigned to 5 KEGG biochemical pathways: cellular processes, environmental information processing, genetic information processing, metabolism and organism system. The x-axis represents the proportion of genes annotated under the pathway occupy the total number of genes being annotated and the y-axis indicates the name of metabolic pathway of KEGG.

Supplementary Table 5. DEGs statistics in different cDNA libraries during pink tea flower development

Formatted: Font: 10 pt, Italic

Formatted: Font: Italic

#Parameters	group	DEGs_total	DEGs_up	DEGs_down
**software:** DESeq2_EBSeq **standard:** FDR=0.01 FC=2	BTP11&BTP12&BTP13_vs_BTP21&BTP22&BTP23	946	513	433
	BTP11&BTP12&BTP13_vs_BTP31&BTP32&BTP33	4272	1763	2509
	BTP11&BTP12&BTP13_vs_BTP41&BTP42&BTP43	9563	4257	5306
	BTP11&BTP12&BTP13_vs_BTP51&BTP52&BTP53	9087	4062	5025
	BTP21&BTP22&BTP23_vs_BTP31&BTP32&BTP33	168	69	99
	BTP21&BTP22&BTP23_vs_BTP41&BTP42&BTP43	6566	3180	3386
	BTP21&BTP22&BTP23_vs_BTP51&BTP52&BTP53	6691	3163	3528

	BTP31&BTP32&BTP33_vs_BTP51&BTP52&BTP53	4766	2189	2577
	BTP32&BTP33&BTP31_vs_BTP42&BTP41&BTP43	4211	2022	2189
	BTP41&BTP42&BTP43_vs_BTP51&BTP52&BTP53	41	28	13

Supplementary Table 6 Primer sequences for qRT-PCR analysis

Gene	Gene no. in Genome/Unigene ID	Forward primer (5′to 3′)	Reverse primer (5′to 3′)	product (bp)	Tm（℃）
LAR-like	CSng5035	AGACGGACGGACAGACAAAG	GGAGTGGTGGAAGGACCGTA	176	60
FLS	CSA003707	CATGTACCCACCATGCCCAC	GAAGACCCGGAACGTCATTG	104	60
FLS	CSA008358	CGGGACTGGGGGATATTTCAG	TAGACCCAGGAGGCTTAGCA	136	60
LDOX1	CSA011508	GGCCTAAGACACCATCCGAC	TAGTCCCAAGCCGAGTGAGA	107	60
CHS	CSA029707	GATATCTCCCTTGCCACCGA	CGAGGAGCTTGGTGAGTTGG	164	60
CHS	CSA029773	GCCCAAACCACTCTCCTTGA	AGACAAGCCCAGGAACACAC	100	60
F3H-like	CSA004930	GAAGAGCCAATCACGTTCGC	GATTTCCAGCTTGGCCTTCAA	138	60
DFR	Csng45659	TCGGTGTATTGGTGCCTTGC	GGCAAACTAAAACTCCGGTCA	170	60
CHI	CSA023536	CACGGCTATCGGAGTGTACC	CTTTTACCACGTGCGGAGAC	132	60

Formatted: Font: 10 pt, Italic

Formatted: Font: Palatino Linotype, 10 pt, Italic

CHS	CSA024718	GGCTGCAACAAAGGCAATCA	GATGGGCGAAGACCGAGTAG	135	60
CHS	CSA029772	CAATCACGTTCGCGGATATGT	ATTTCCAGCTTGGCCTTCAAA	130	60
ANR	CSA011986	AGCACTAAAGGGTTCAGGCA	AATTGCCTCTGGATCCTCGG	145	60
β-actin	KJ946252.1	TGCTGGCCGTGATCTAACAG	TGCTCGTAGTCAAGAGCGAC	135	60

Supplementary Table 7 Flavonoid-related gene module (turquoise module) by WGCNA analysis

Formatted: Font: 10 pt, Italic

Formatted: Font: Palatino Linotype, Italic

Biological process in GO enrichment	Gene no. in Genome/Unigene ID	Gene name
	CSA023536	chalcone synthase(CHS)
	CSA029772	chalcone synthase(CHS)
	CSA029707	chalcone synthase(CHS)
	CSA024718	chalcone synthase(CHS)
	CSA029773	chalcone synthase(CHS)
	CSA004930	flavanone-dioxygenase-like (F3H-like)
	CSA023536	chalcone isomerase (CHI)
Biological Process: flavonoid	CSA008358	flavonol synthase (FLS)
biosynthetic process (GO:0009813)	Csng45659	dihydroflavonol-4-reductase (DFR)
Biological Process: proanthocyanidin biosynthetic (GO:0010023) process	CSA011508	leucoanthocyanidin dioxygenase isoform 1(LDOX1)
